# XerC Is Required for the Repair of Antibiotic- and Immune-Mediated DNA Damage in Staphylococcus aureus

**DOI:** 10.1128/aac.01206-22

**Published:** 2023-02-21

**Authors:** Elizabeth V. K. Ledger, Katie Lau, Edward W. Tate, Andrew M. Edwards

**Affiliations:** a MRC Centre for Molecular Bacteriology and Infection, Imperial College London, London, United Kingdom; b Department of Chemistry, Molecular Sciences Research Hub, Imperial College London, London, United Kingdom

**Keywords:** *Staphylococcus aureus*, DNA repair, XerC, antibiotics, immune response, DNA, SOS system, *Staphylococcus*, neutrophils, repair

## Abstract

To survive in the host environment, pathogenic bacteria need to be able to repair DNA damage caused by both antibiotics and the immune system. The SOS response is a key bacterial pathway to repair DNA double-strand breaks and may therefore be a good target for novel therapeutics to sensitize bacteria to antibiotics and the immune response. However, the genes required for the SOS response in Staphylococcus aureus have not been fully established. Therefore, we carried out a screen of mutants involved in various DNA repair pathways to understand which were required for induction of the SOS response. This led to the identification of 16 genes that may play a role in SOS response induction and, of these, 3 that affected the susceptibility of S. aureus to ciprofloxacin. Further characterization revealed that, in addition to ciprofloxacin, loss of the tyrosine recombinase XerC increased the susceptibility of S. aureus to various classes of antibiotics, as well as to host immune defenses. Therefore, the inhibition of XerC may be a viable therapeutic approach to sensitize S. aureus to both antibiotics and the immune response.

## INTRODUCTION

Staphylococcus aureus is a leading cause of infections, ranging from those affecting the skin and soft tissues to life-threatening invasive diseases such as bacteremia and endocarditis (1). Treatment of these infections can be challenging, especially when they are caused by multidrug-resistant strains. However, even in the case of infections caused by drug-susceptible strains, high rates of relapse and development of chronic infections are observed (2, 3).

For S. aureus to survive in the host and establish an infection, it must be able to survive damage caused by both the immune system and antibiotics. One of the main immune cell types used by the host to control S. aureus infections is neutrophils, which typically kill S. aureus via antimicrobial peptides and reactive oxygen species (ROS) released during the oxidative burst (4). These ROS damage many cellular components, including nucleic acids, which results in DNA double-strand breaks that are lethal if not repaired by the bacterium (5, 6). Some clinically important antibiotics directly target DNA synthesis and replication, for example, fluoroquinolones, which inhibit DNA gyrase, or co-trimoxazole, which inhibits tetrahydrofolate synthesis, thereby causing stalled DNA replication. In addition, other classes of bactericidal antibiotics, including beta-lactams and daptomycin, cause DNA damage indirectly via the production of ROS (7–9). Therefore, it is well established that S. aureus needs to be able to repair DNA damage to survive inside the host.

The main pathway used by bacteria to repair DNA damage is the SOS response, which regulates expression of genes responsible for DNA repair (10). In S. aureus, DNA double-strand breaks are processed by the helicase/nuclease activity of RexAB (a member of the RecBCD/AddAB family) to generate single-stranded DNA (11). This is then bound by RecA, forming a nucleoprotein filament that triggers the autocleavage of LexA, a transcriptional repressor, derepressing expression of genes in the SOS regulon (10). In S. aureus, the SOS regulon consists of 16 genes, including *lexA* and *recA*, and several genes involved in repairing various types of DNA damage, including nucleotide excision repair and the processing of stalled replication forks (12). Many antibiotics have been shown to induce the SOS response in S. aureus, including directly DNA-damaging agents, such as ciprofloxacin, co-trimoxazole, and nitrofurantoin, and also antibiotics with other targets, including daptomycin, oxacillin, and chloramphenicol (9, 12–14). In line with this, a mutant defective for *rexB* was unable to induce the SOS response when exposed to these agents and showed increased antibiotic susceptibility (9).

In addition to regulating DNA repair, the SOS response plays a key role in the emergence of antibiotic resistance. One of the genes most strongly expressed in response to induction of the SOS response is *umuC*, which encodes an error-prone polymerase (12, 15). Therefore, activation of the SOS response leads to an increase in the mutation rate, promoting the emergence of antibiotic resistance. As well as this, induction of the SOS response activates prophages present in the S. aureus genome, leading to the dissemination of virulence and antibiotic resistance genes via horizontal gene transfer (16).

Since the SOS response promotes survival of both antibiotics and the immune response as well as increasing the emergence of antibiotic resistance and the spread of virulence genes, its inhibition is an attractive therapeutic target to sensitize S. aureus to the action of antibiotics and the immune response. In support of this, several inhibitors of RecBCD/AddAB family proteins have been identified, including the Gam protein of bacteriophage lambda, which sensitizes Escherichia coli to ciprofloxacin (17, 18). However, due to poor pharmacokinetic/pharmacodynamic properties and/or toxicity, none are suitable therapeutics (18).

Unfortunately, while the SOS response has been well characterized in several model bacteria, the response of S. aureus differs significantly from these and is poorly understood. For example, the SOS regulons of E. coli and Bacillus subtilis comprise 43 and 63 genes, respectively, many more than the 16 in S. aureus (10). Additionally, which genes are required for induction of the SOS response, and may therefore constitute good therapeutic targets, is not well understood. Therefore, to address this, we carried out a screen of genes previously identified to play a role in DNA repair to determine which were important for inducing the SOS response and modulating susceptibility to antibiotics and the immune response.

## RESULTS

### XerC and XseA are required for the repair of ciprofloxacin-mediated DNA damage.

To establish which S. aureus genes were required for the SOS response, we identified and screened 54 mutants from the Network on Antimicrobial Resistance in Staphylococcus aureus (NARSA) transposon library with transposons inserted in genes that had previously been described to play or proposed to play a role in DNA repair (see Table S1 in the supplemental material) (19). Where two genes contributed to a single protein complex (e.g., RexAB), only one gene was included in the screen. To measure the SOS response in each of these mutants, we used a reporter plasmid consisting of the *recA* promoter upstream of *gfp*, which was transduced into the JE2 wild-type (WT) strain and each transposon mutant (13). We have previously validated this reporter, showing dose-dependent induction of green fluorescent protein (GFP) production in response to well-established triggers of the SOS response, including ciprofloxacin, mitomycin C, and oxidative stress due to paraquat (9, 11, 13). Further validation comes from an absence or greatly reduced GFP production in mutants unable to process DNA to single strands, which is a prerequisite for SOS induction (9, 13).

**FIG 1:**
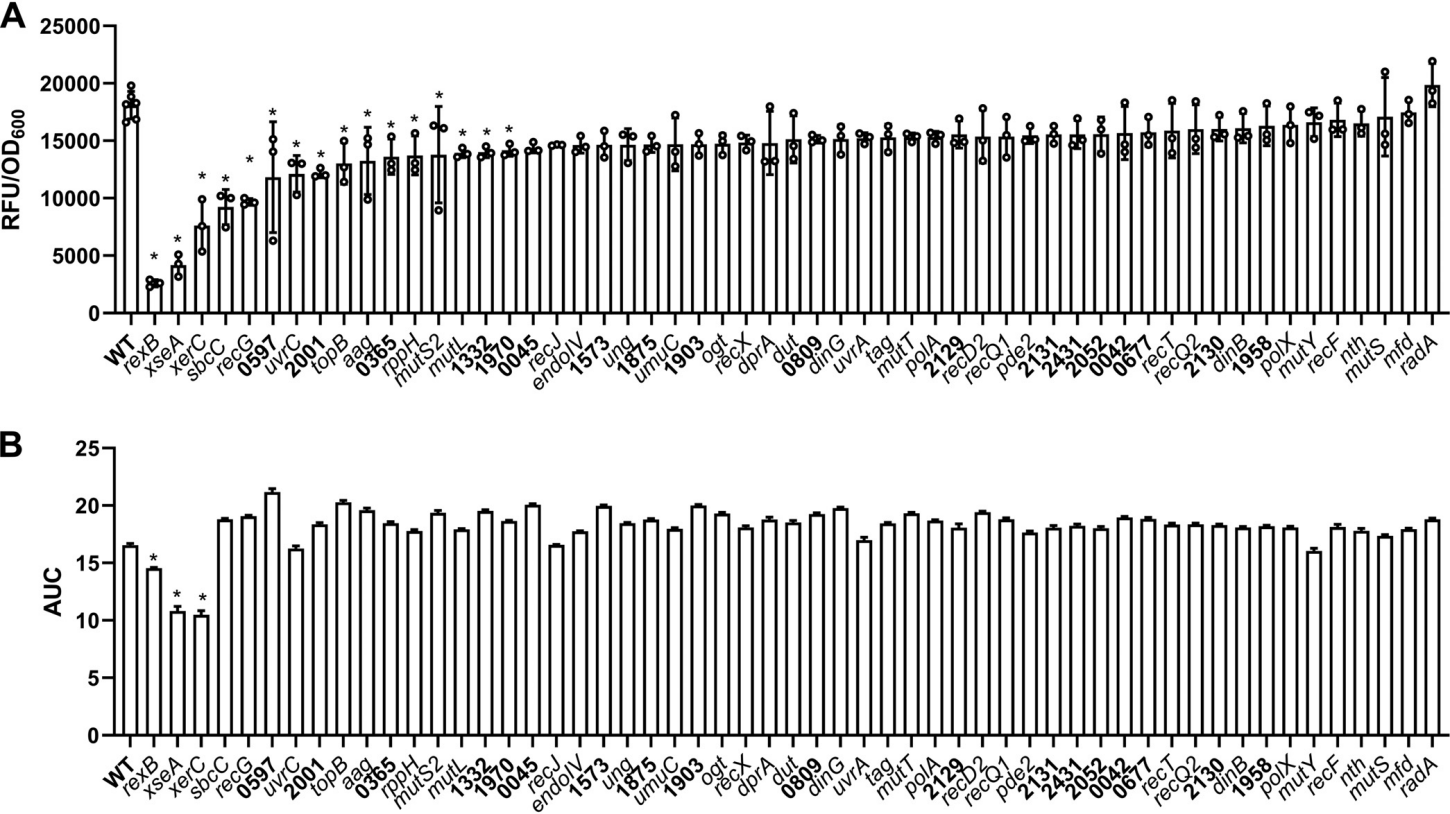
XerC and XseA are required for repair of ciprofloxacin-mediated DNA damage. S. aureus USA300 JE2 WT and mutants defective in various DNA repair genes containing the P*recA-gfp* SOS response reporter plasmid were exposed to 16 μg mL^−1^ ciprofloxacin for 17 h. (A) Induction of the SOS response was determined by measuring GFP fluorescence, and the peak fluorescence value of each strain (adjusted for cell density) was plotted. (B) The OD_600_ was measured every 15 min throughout the 17 h ciprofloxacin exposure, and the area under the curve (AUC) was plotted. Data in panel A represent the mean ± standard deviation of at least three independent experiments, and data in panel B represent the mean ± standard error of at least three independent experiments. In each case, data were analyzed by one-way analysis of variance (ANOVA) with Dunnett’s *post hoc* test (*, *P* < 0.05; WT versus mutant).

Strains containing the reporter construct were then exposed to a range of therapeutically relevant concentrations of the DNA-damaging antibiotic ciprofloxacin (0 to 16 μg mL^−1^) and the SOS response measured over time (Fig. S1). The peak fluorescence value of each strain at 16 μg mL^−1^ ciprofloxacin was plotted to enable comparison between strains (Fig. 1A). This led to the identification of 16 mutants with a significantly reduced SOS response compared to the WT strain, including the *rexB*::Tn mutant, which has been previously identified as essential for induction of the SOS response in S. aureus and thus validated our approach.

Second, we generated growth curves of each strain during a 17 h exposure to 16 μg mL^−1^ ciprofloxacin and plotted the area under the curve to give a measure of the susceptibility of each mutant to the antibiotic (Fig. S2). This demonstrated that three strains, *rexB*::Tn, *xerC*::Tn, and *xseA*::Tn, were more susceptible to ciprofloxacin than the WT strain (Fig. 1B).

Taken together, we identified 16 mutants with an impaired ability to induce the SOS response on exposure to ciprofloxacin, and of these, 3 mutants were also more susceptible to ciprofloxacin, suggesting that they may be good targets to sensitize S. aureus to DNA-damaging antibiotics.

### XerC is required for the SOS response induced by various DNA-damaging agents.

In addition to ciprofloxacin, many other DNA-damaging agents also induce the SOS response. Therefore, we next aimed to determine whether the impaired SOS induction occurred in response to various agents which cause DNA damage via diverse mechanisms or whether it was unique to ciprofloxacin. As we have previously demonstrated that RexB is required for induction of the SOS response and preliminary experiments with the *xseA*::Tn strain produced inconsistent results, we focused on the role of XerC (11).

**FIG 2:**
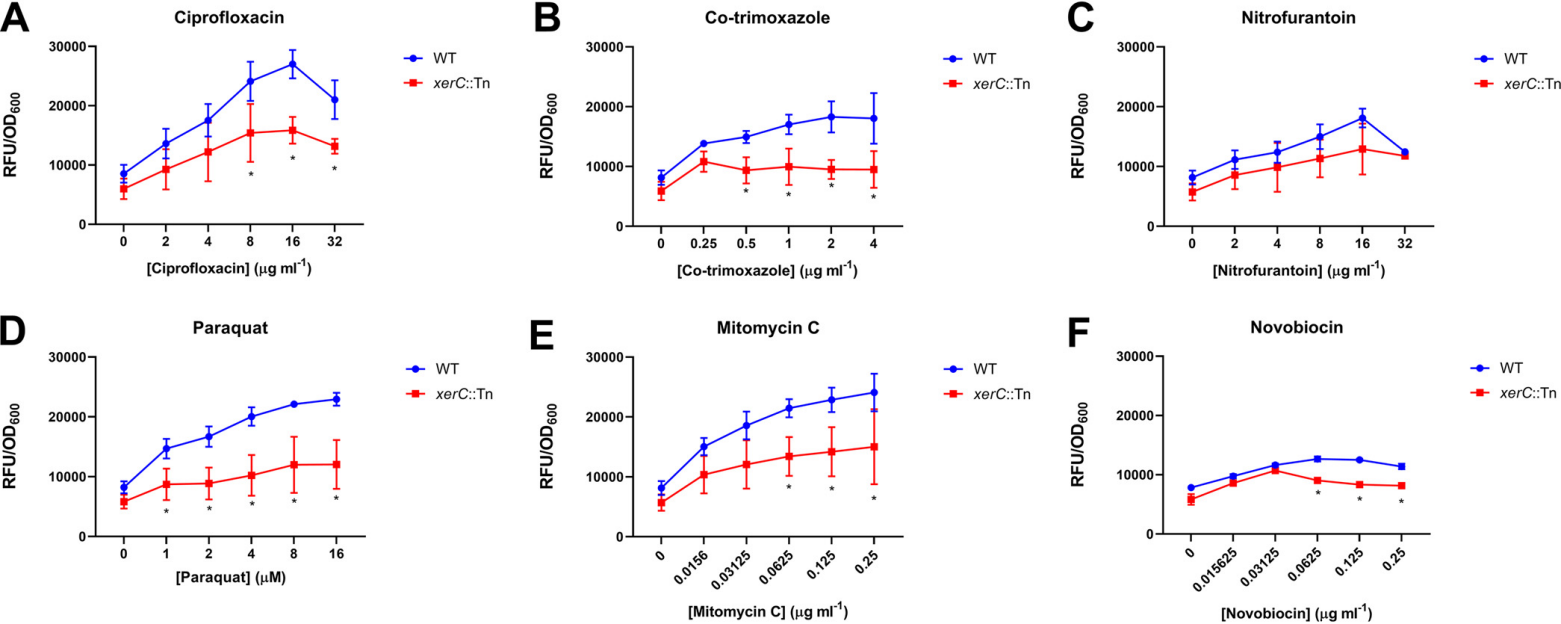
XerC is required for SOS response induced by various DNA-damaging agents. S. aureus USA300 JE2 WT and the *xerC*::Tn mutant containing the P*recA-gfp* SOS response reporter plasmid were exposed to indicated concentrations of ciprofloxacin (A), co-trimoxazole (B), nitrofurantoin (C), paraquat (D), mitomycin C (E), or novobiocin (F) before the peak fluorescence value of each strain (adjusted for cell density) was plotted. Data represent the mean ± standard deviation of three independent experiments and were analyzed by two-way ANOVA with Dunnett’s *post hoc* test (*, *P* < 0.05; WT versus mutant at indicated concentration).

To do this, we exposed the WT and *xerC*::Tn mutant to a range of concentrations of ciprofloxacin, co-trimoxazole, nitrofurantoin, mitomycin C, and paraquat, which generates superoxide radicals, while measuring the SOS response using the P*recA-gfp* reporter system. In addition, the strains were exposed to novobiocin, an inhibitor of DNA gyrase which has previously been reported not to induce the SOS response (20). Exposure to each of the DNA-damaging agents led to a dose-dependent increase in induction of the SOS response in the WT strain, although this was very weak for novobiocin (Fig. 2A to F). However, with the exception of nitrofurantoin, the SOS response of the *xerC*::Tn mutant strain was significantly lower than that of the WT strain at higher concentrations of the genotoxic agents, indicating that XerC is required for maximal SOS induction in response to diverse types of DNA damage (Fig. 2A to F).

Unfortunately, despite several attempts, we were unable to complement the *xerC*::Tn P*recA-gfp* reporter strain with the wild-type *xerC* gene or transform the complemented strain with the reporter construct. We hypothesize this is due to plasmid incompatibility or issues with the dual-antibiotic selection needed to maintain both plasmids.

In many species of bacteria, XerC has been implicated in the monomerization of plasmids, enabling the stable segregation of plasmids during cell division (21). As our reporter system was plasmid based, we checked whether an alternative explanation for the reduced SOS response of the mutant strain was a loss of the reporter system during the assay. To do this, we plated bacteria after carrying out the SOS reporter assay onto tryptic soy agar (TSA) or TSA supplemented with kanamycin and determined the percentage of CFU retaining the reporter plasmid. This demonstrated that no loss of the reporter plasmid from bacteria occurred during the assay in either the WT strain or the *xerC*::Tn mutant (Fig. S3).

**FIG 3:**
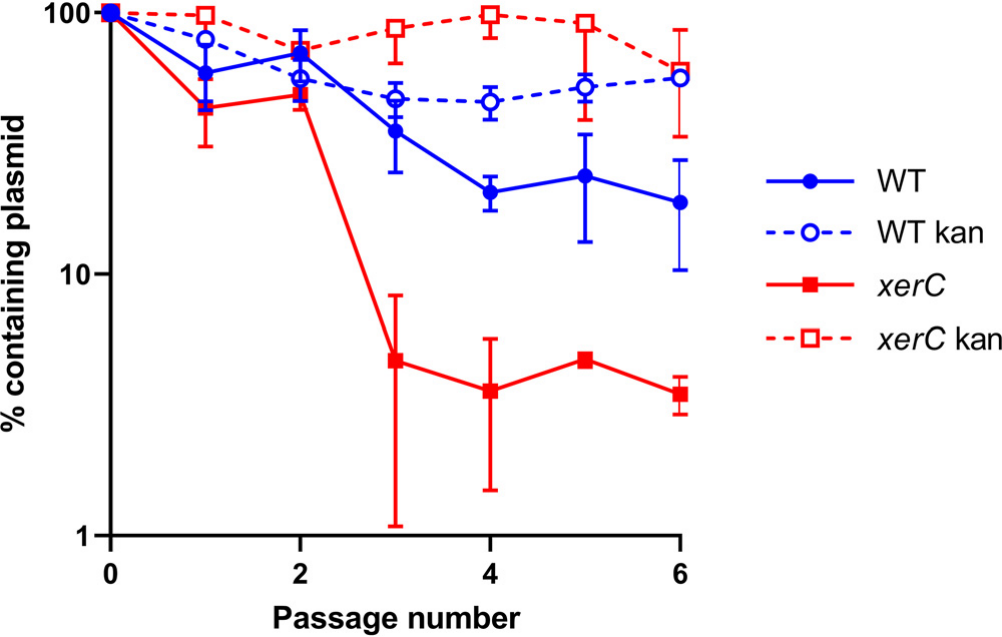
XerC is required for plasmid stability in the absence of selection pressure. S. aureus USA300 JE2 WT and *xerC*::Tn containing the P*recA-gfp* reporter plasmids underwent 6 passages in TSB or TSB supplemented with 90 μg mL^−1^ kanamycin. After each passage, strains were plated on TSA and TSA supplemented with 90 μg mL^−1^ kanamycin, and the percentage of colonies containing the plasmid was determined. Data represent the mean ± standard deviation of three independent experiments.

Next, we investigated whether XerC may play a role in separating plasmid multimers, as has been described in other bacteria (22–24). To do this, we carried out a passage experiment, repeatedly subculturing both the WT and *xerC*::Tn strains carrying the P*recA-gfp* reporter plasmid in either the presence of kanamycin to select for the plasmid or the absence of selection pressure. Each passage consisted of a 1:1,000 dilution of the bacterial cultures, enabling approximately 10 cell divisions each passage. This was repeated 6 times, resulting in approximately 60 generations occurring during the assay. As expected, in the presence of kanamycin, there was no significant loss of plasmid throughout the experiments in either the WT or the *xerC*::Tn strain (Fig. 3). In contrast, in the absence of selection, the plasmid was slowly lost from the WT strain, while between 20 and 30 generations, there was a significant reduction of the number of *xerC*::Tn bacteria that carried the plasmid, with over 95% of colonies having lost the plasmid by 30 generations (Fig. 3).

### Lack of XerC-mediated DNA repair sensitizes S. aureus to various classes of antibiotics.

Next, we aimed to determine whether XerC-mediated DNA repair affected susceptibility of S. aureus to a range of antibiotics. We measured the susceptibility of the WT and *xerC*::Tn strains to a panel of antibiotics, including several which directly damage DNA, as well as cell wall-targeting antibiotics and protein synthesis inhibitors, some of which have been reported to damage DNA indirectly, for example, via the production of ROS.

**FIG 4:**
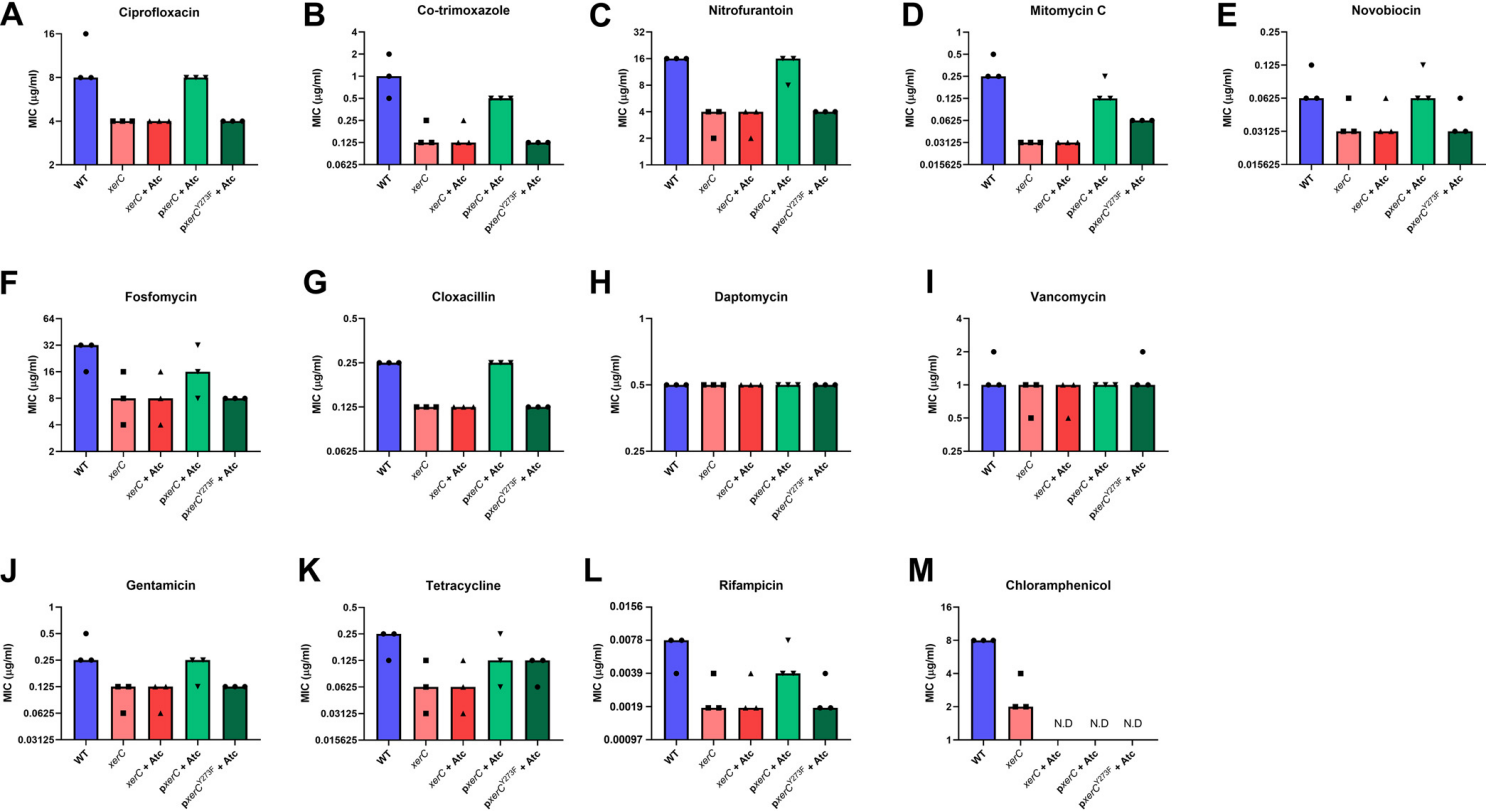
Lack of XerC-mediated DNA repair increases susceptibility of S. aureus to various classes of antibiotic. MICs of S. aureus USA300 JE2 WT, the *xerC*::Tn mutant, and the *xerC*::Tn mutant complemented with p*xerC* or p*xerC*^Y273F^ to ciprofloxacin (A), co-trimoxazole (B), nitrofurantoin (C), mitomycin C (D), novobiocin (E), fosfomycin (F), cloxacillin (G), daptomycin (H), vancomycin (I), gentamicin (J), tetracycline (K), rifampicin (L), and chloramphenicol (M). Data represent the median of three independent experiments. N.D., not determined; Atc, anhydrotetracycline.

Susceptibility to five directly DNA-damaging antimicrobials, ciprofloxacin, co-trimoxazole, nitrofurantoin, mitomycin C, and novobiocin, was determined by establishing their minimum inhibitory concentrations (MICs). In each case, the *xerC*::Tn mutant was more susceptible than WT, with the largest increases in susceptibility (8-fold) being observed with co-trimoxazole and mitomycin C, followed by 4-fold reductions in MIC for the XerC-deficient strain for nitrofurantoin, fosfomycin, rifampicin, tetracycline, and chloramphenicol (Fig. 4). The *xerC*::Tn mutant was 2-fold more susceptible to the DNA-damaging antibiotics ciprofloxacin and novobiocin, which both target DNA gyrase (Fig. 4A and E). We also found that S. aureus resistant to novobiocin (MIC = 8 μg mL^−1^) was 2-fold more susceptible to this antibiotic when XerC was absent (MIC = 4 μg mL^−1^).

In addition to DNA-damaging antibiotics, the *xerC*::Tn mutant was also more susceptible to the cell wall synthesis inhibitors fosfomycin and cloxacillin, but not daptomycin or vancomycin, which target the cell envelope, and each of the four protein synthesis inhibitors examined, gentamicin, tetracycline, rifampicin, and chloramphenicol (Fig. 4).

Next, we aimed to confirm that this was due to the DNA-repair ability of XerC. XerC is a tyrosine-type site-specific recombinase, which uses an active site tyrosine (Y273) to break and rejoin DNA at a specific sequence (25). To do this, we complemented *xerC*::Tn with either WT *xerC* or *xerC*^Y273F^, a catalytically inactive version with a mutated active site tyrosine to abolish the recombination activity of XerC, under the control of a tetracycline-inducible promoter (26). The plasmid used for complementation conferred chloramphenicol resistance, precluding further testing of whether XerC contributed to chloramphenicol susceptibility. In each case, complementation with the WT copy of *xerC* at least partially restored antibiotic susceptibility to WT levels (Fig. 4). However, complementation with *xerC*^Y273F^ did not, indicating that the increased sensitivity of the *xerC*::Tn mutant was due to its lack of DNA repair ability (Fig. 4).

Next, we attempted to transduce the *xerC*::Tn mutation into other strains to confirm the phenotype. However, despite repeated attempts into multiple strains from distinct genetic backgrounds, we were unable to do this, possibly due to the important role XerC plays in DNA recombination (25, 27).

**FIG 5:**
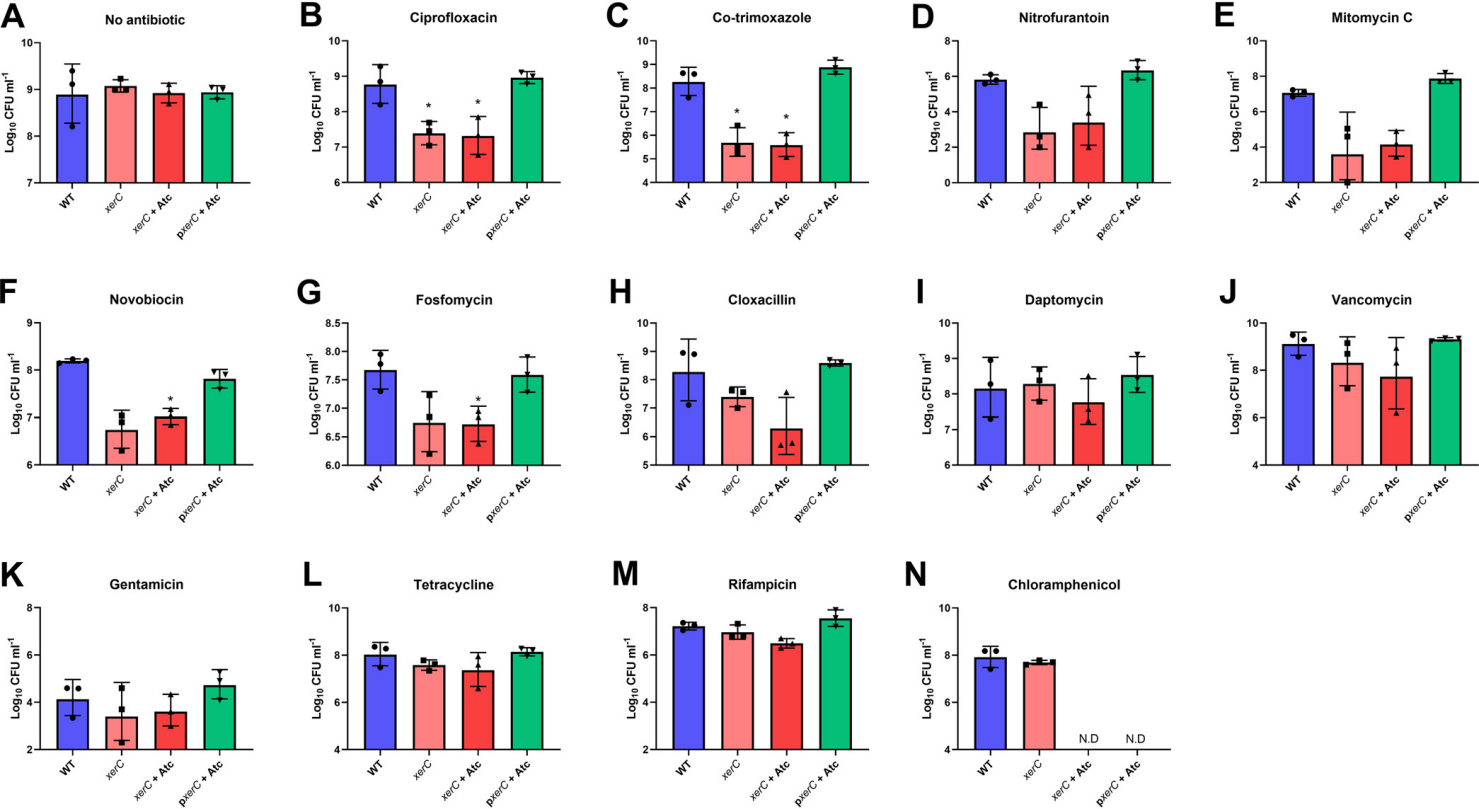
Lack of XerC sensitizes S. aureus to killing by certain classes of antibiotics. Log_10_ CFU mL^−1^ of S. aureus USA300 JE2 WT, the *xerC*::Tn mutant, and the *xerC*::Tn mutant complemented with p*xerC* after a 6 h exposure of 10^8^ CFU mL^−1^ to no antibiotic (A), 40 μg mL^−1^ ciprofloxacin (B), 5 μg mL^−1^ co-trimoxazole (C), 80 μg mL^−1^ nitrofurantoin (D), 1.25 μg mL^−1^ mitomycin C (E), 10 μg mL^−1^ novobiocin (F), 160 μg mL^−1^ fosfomycin (G), 1.25 μg mL^−1^ cloxacillin (H), 2.5 μg mL^−1^ daptomycin (I), 5 μg mL^−1^ vancomycin (J), 2.5 μg mL^−1^ gentamicin (K), 2.5 μg mL^−1^ tetracycline (L), 0.038 μg mL^−1^ rifampicin (M), and 40 μg mL^−1^ chloramphenicol (N). Data represent the geometric mean ± geometric standard deviation of three independent experiments. Data were analyzed by one-way ANOVA with Dunnett’s *post hoc* test (*, *P* < 0.05, WT versus mutant). N.D., not determined; Atc, anhydrotetracycline.

Finally, we measured the survival of bacteria exposed to antibiotics as a second measure of drug susceptibility. To do this, 10^8^ CFU mL^−1^ of each strain was exposed to 5× the MIC of the WT strain or no antibiotic for 6 h before surviving bacteria were enumerated. In the absence of antibiotic, each strain grew 10-fold to 10^9^ CFU mL^−1^ (Fig. 5A). In agreement with the MIC data, the *xerC*::Tn mutant was killed to a greater extent than the WT strain on exposure to each of the directly DNA-damaging antibiotics (Fig. 5). The biggest effects were seen for ciprofloxacin, co-trimoxazole, nitrofurantoin, and mitomycin C, with >50-fold increased killing of the *xerC*::Tn mutant (Fig. 5B to E). Similarly, greater killing of the mutant strain was observed on exposure to novobiovin, fosfomycin, and cloxacillin as well (Fig. 5G to H). In line with the observation that the WT and mutant strains had the same MICs to vancomycin and daptomycin, no differences in killing at 6 h were seen with these antibiotics (Fig. 5I to J). However, in contrast to the MIC data, no differences in survival between the strains were observed with any of the protein synthesis inhibitors (Fig. 5K to N).

### Lack of XerC sensitizes S. aureus to immune-mediated killing.

In the host, S. aureus DNA damage is not only caused by antibiotic exposure but also by the immune system, particularly by reactive oxygen species produced by neutrophils. Therefore, we used a whole human blood model to test whether XerC was required for surviving the DNA damage since previous work from our group and others have shown that S. aureus in blood is rapidly phagocytosed by neutrophils and exposed to the oxidative burst (11, 28).

**FIG 6:**
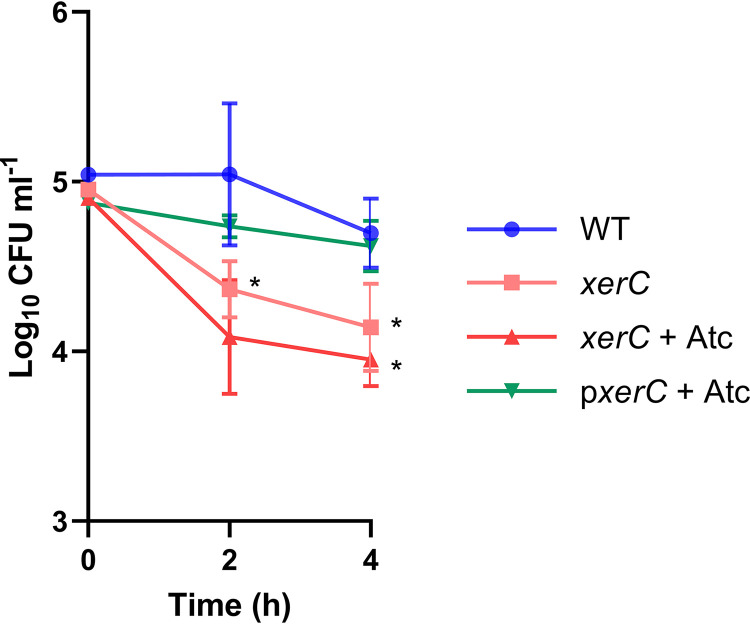
Lack of XerC sensitizes S. aureus to killing by human blood. Log_10_ CFU mL^−1^ of S. aureus USA300 JE2 WT, the *xerC*::Tn mutant and complemented mutant during a 4 h of incubation in whole human blood. Data represent the geometric mean ± geometric standard deviation of three independent experiments. Data were analyzed by two-way ANOVA with Dunnett’s *post hoc* test (*, *P* < 0.05, WT versus mutant). Atc, anhydrotetracycline.

The WT strain demonstrated high levels of survival on exposure to whole blood from healthy human donors (Fig. 6). In contrast, significantly lower survival of the *xerC*::Tn mutant strain was observed, and this defect in survival was fully complemented by expression of *xerC* on a plasmid (Fig. 6).

Taken together, XerC is required for induction of the SOS response, with the result that a mutant defective in *xerC* is more susceptible to a range of DNA-damaging antibiotics and immune-mediated killing.

## DISCUSSION

Treatment of infections caused by S. aureus is challenging due to increasing rates of antibiotic resistance. In addition to discovering novel antibiotics, an alternative approach is to develop compounds which sensitize bacteria to existing antibiotics. One such approach is to sensitize S. aureus to DNA-damaging antibiotics by inhibiting bacterial DNA repair via targeting the SOS response pathway (18). Unfortunately, this approach has been hindered by S. aureus having a poorly characterized SOS response, meaning that the potentially best proteins to target have not yet been identified. Therefore, to address this, we measured the ability of 54 mutants in various DNA repair pathways to induce the SOS response on exposure to ciprofloxacin.

In S. aureus, DNA double-strand breaks are processed by the RexAB enzyme complex to generate a piece of single-stranded DNA that is bound by RecA, resulting in the autocleavage of the LexA repressor and activation of the SOS response (11, 29). In agreement with this, our screen of mutants defective for various DNA repair proteins identified that lack of RexB significantly reduced SOS induction, validating our screening approach. Through our screen, we identified two additional genes, *xerC* and *xseA*, where disruption reduced SOS induction and increased ciprofloxacin susceptibility. *xseA*, together with *xseB* (not included in the screen), encodes DNA exonuclease VII, which degrades single-stranded DNA (30). In E. coli, it has been suggested that this complex processes DNA double-strand breaks to generate blunt DNA ends, which can then be recognized by RecBCD, triggering the SOS response (31). *xerC* encodes a tyrosine recombinase, which, together with *xerD* (not included in the screen), is important for resolving the DNA multimers which form during DNA replication and repair (32). The reason why XerC is required for optimal induction of the SOS response in S. aureus is currently unclear and contrasts with work in E. coli demonstrating that the absence of *xerC* did not affect SOS induction (33). However, this finding further emphasizes differences in the mechanisms of induction of the SOS response between S. aureus and much more well-characterized organisms such as E. coli.

The absence of a *xerD* mutant from the NARSA library prevented analysis of the role of this gene and its product in repair of DNA damage caused by antibiotics or the immune system and comparison with XerC. It is not clear why there is no *xerD*::Tn mutant. Previous work using transposon insertion sequencing (Tn-Seq) of several different S. aureus lineages indicated that XerD is essential in some strains but not others, although the data were inconclusive for the USA300 strain used here (34). Another Tn-Seq study showed that XerD was not essential in USA300 (35), but other work suggested compromised fitness under laboratory conditions of the NCTC 8325 strain (36). Of note, the latter study found that *xerC* was essential in S. aureus NCTC 8325, indicating that the essentially of both XerC and XerD may be strain dependent (36).

As well as *xseA*, *xerC*, and *rexB*, we also identified 15 genes where disruption significantly reduced SOS induction but had no effect on ciprofloxacin susceptibility. In these cases, the SOS response was not as impaired as the mutants where DNA repair was inhibited, and so, although the SOS response was reduced, there may have been a sufficient level to enable DNA repair. Alternatively, these mutants may upregulate alternative DNA repair pathways to compensate for the lack of SOS response induction. This finding has implications for the design of screens to identify new inhibitors of DNA repair, demonstrating the importance of characterizing susceptibility to antibiotics as well as the SOS response.

In addition to resolving chromosomal multimers, XerC plays a key role in monomerizing plasmids in many bacterial species, including E. coli, Salmonella enterica serovar Typhimurium, Klebsiella pneumoniae, and Acinetobacter baumannii (22–24). Our finding that XerC was required for preventing plasmid loss in S. aureus indicates that XerC may play a similar role in this bacterium. This may have important implications for the spread of antibiotic resistance and virulence genes, as in S. aureus, these are frequently located on plasmids (37). However, a limitation of this work is that these plasmid segregation assays were performed with an E. coli-S. aureus shuttle vector, which may not be representative of plasmids found in clinical S. aureus strains. However, while large plasmids typically encode their own machinery for monomerization, smaller plasmids found in clinical S. aureus strains do not and, instead, rely on bacterially encoded systems (38). Therefore, in some cases, therapeutic inhibition of XerC may lead to a loss of antibiotic resistance, sensitizing S. aureus to antibiotics.

Beyond the loss of plasmid-mediated resistance, very few studies have examined the direct role of XerC in antibiotic susceptibility, especially in S. aureus. A previous screen of the NARSA library identified the mutant defective for *xerC* was more susceptible to ciprofloxacin and gentamicin but not to oxacillin, linezolid, fosfomycin, daptomycin, mupirocin, or vancomycin (39). Increased ciprofloxacin susceptibility has also been reported for E. coli and P. aeruginosa *xerC* mutants (40, 41). However, our finding that the S. aureus *xerC* mutant is more susceptible to antibiotics from a range of classes fits with the growing evidence that many antibiotics lead to DNA damage via the production of ROS (42). The reason why XerC is required for repair of antibiotic-induced DNA damage is currently unknown. However, one hypothesis is that when bacteria repair DNA damage through homologous recombination, DNA dimers are produced, which must be resolved by the activity of XerCD to enable subsequent DNA replication to occur and for correct DNA segregation during cell division (38). However, this remains to be demonstrated experimentally.

Finally, in agreement with data showing that neutrophils cause DNA double-strand breaks which are repaired via the SOS response, the *xerC*::Tn mutant showed significantly reduced survival in a whole human blood model (11). This finding agrees with previous work indicating that XerD contributes to survival of S. aureus NCTC 8325 in abscesses and the bloodstream of mice (36) and adds to our appreciation of the importance of DNA repair pathways inside the host. It also provides a possible explanation for the reduced virulence of the *xerC* mutants observed in murine bacteremia and pyelonephritis models (27, 43). Additionally, it may suggest that chemical inhibition of XerC could be a viable monotherapeutic approach, as it may enhance bacterial killing by the immune system.

In summary, characterization of the SOS response in S. aureus has led to the identification of XerC as a key protein required for the induction of this pathway. The benefits of targeting this protein complex are numerous, as it is required for survival inside the host, meaning that inhibitors of XerC may have activity alone, reducing bacterial virulence and enhancing immune-mediated killing. In addition, they may potentiate various classes of existing antibiotics, including directly DNA-damaging antibiotics such as ciprofloxacin and co-trimoxazole, and also cell wall-targeting agents and protein synthesis inhibitors. Finally, inhibitors of XerC may reduce rates of antibiotic resistance by preventing the segregation of plasmids carrying antibiotic resistance genes or by blocking the SOS response. As well as reducing the mutation rate and therefore the emergence of spontaneous resistance, this also suppresses the activation of prophages, slowing the dissemination of antibiotic resistance and virulence genes.

## MATERIALS AND METHODS

### Bacterial strains and growth conditions.

The bacterial strains used in this study are shown in Table S2. S. aureus was grown in tryptic soy broth (TSB) for 16 h at 37°C with shaking (180 rpm). When required, TSB was supplemented with erythromycin (10 μg mL^−1^), kanamycin (90 μg mL^−1^), or chloramphenicol (5 μg mL^−1^). For strains complemented with p*itet*, media were supplemented with 100 ng mL^−1^ anhydrotetracycline (Atc) to induce expression.

### Construction of strains.

The P*recA-gfp* reporter plasmid was transduced from JE2 WT P*recA-gfp* (13) into mutants obtained from the NARSA library using φ11, as previously described (44).

Complementation of the *xerC*::Tn mutant was carried out using p*itet* (26). The *xerC* gene was amplified from JE2 WT genomic DNA using *xerC*_Fw and *xerC*_Rev primers (see Table S3 in the supplemental material). To construct p*itet*-*xerC*, the PCR product and the p*itet* vector were digested with avrII and pmeI, ligated using T4 ligase, and transformed into E. coli DC10B. p*itet-xerC* was then electroporated into RN4220 and then transduced into the *xerC*::Tn mutant using φ11. To construct *xerC*::Tn p*itet-xerC*^Y273F^, site-directed mutagenesis was performed using p*itet-xerC* as a template, the *xerC-*Y273F_Fw and *xerC*-Y273F_Rev primers (Table S3), and the Q5 site-directed mutagenesis kit according to the manufacturer’s instructions. From E. coli, p*itet-xerC*^Y273F^ was transformed into RN4220 and then transduced into the *xerC*::Tn mutant using φ11.

A novobiocin-resistant strain was generated by setting up repeated MIC assays (described below) starting with an inoculum of JE2 WT. Each day, the MIC assay was inoculated with bacteria from the well containing the highest antibiotic concentration which supported bacterial growth from the previous day’s MIC assay. Once a strain had been generated with an MIC of 8 μg mL^−1^, a phage lysate of this strain was made using φ11 and used to transduce the novobiocin resistance-conferring mutation into the *xerC*::Tn mutant as previously described (44).

### Measurement of SOS response by fluorescent reporter assay.

Strains containing the P*recA-gfp* reporter plasmid were used to quantify *recA* expression as a measure of induction of the SOS response as previously described (13). Twofold serial dilutions of antibiotic were performed in TSB in black 96-well plates in a final volume of 200 μL. Wells were inoculated with 10^8^ CFU mL^−1^, and plates were incubated for 17 h in a Tecan Infinite M200 Pro plate reader at 37°C with orbital shaking (300 rpm). Every 15 min, fluorescence (excitation, 485 nm; emission, 525 nm) and optical density at 600 nm (OD_600_) were measured. Relative fluorescence unit (RFU) values were divided by OD_600_ at each time point to normalize for changes in cell density which occurred during the assay, and the peak RFU/OD_600_ was plotted.

### Measurements of plasmid stability.

To determine the rate at which the P*recA-gfp* reporter plasmid was lost from the JE2 WT and *xerC*::Tn mutant strains, each of these strains containing the plasmid was passaged six times in the presence or absence of kanamycin selection. Each passage consisted of a 1,000-fold dilution in TSB supplemented or not with 90 μg mL^−1^ kanamycin and then 8 h growth at 37°C with shaking (180 rpm). Cultures were stored overnight between passages at 4°C. After each passage, each culture was serially diluted 10-fold in phosphate-buffered saline (PBS) and plated onto TSA with and without 90 μg mL^−1^ kanamycin to enumerate CFU mL^−1^. The CFU mL^−1^ on TSA plus kanamycin was divided by the CFU mL^−1^ on TSA to calculate the proportion of CFU containing the plasmid.

### Determination of antibiotic MICs.

MICs were determined using a broth microdilution protocol as described previously (45). Twofold serial dilutions of antibiotics were prepared in a final volume of 200 μL TSB in 96-well plates. For daptomycin, 1.25 mM CaCl_2_ was added to the media. Wells were inoculated to 5 × 10^5^ CFU mL^−1^ bacteria and incubated statically for 17 h at 37°C. The MIC was defined as the minimum concentration of antibiotic where no visible growth was observed.

### Antibiotic killing assay.

TSB (3 mL) supplemented with antibiotics at 5× WT MIC was inoculated with 10^8^ CFU mL^−1^ S. aureus. Cultures were incubated at 37°C for 6 h with shaking (180 rpm). For daptomycin, 1.25 mM CaCl_2_ was added to the media. After 0 h and 6 h antibiotic exposure, cultures were serially diluted 10-fold in PBS and plated on TSA to enumerate CFU mL^−1^.

### Whole human blood killing assay.

Ethical approval for drawing and using human blood was obtained from the Regional Ethics Committee and the Imperial NHS Trust Tissue Bank (REC Wales approval no. 12/WA/0196 and ICHTB HTA license no. 12275). Whole human blood was collected in EDTA tubes. Overnight cultures of S. aureus were washed twice in PBS and resuspended at 10^6^ CFU mL^−1^. In a 96-well plate, 90 μL blood was inoculated with 10 μL bacteria to give a final inoculum of 10^5^ CFU mL^−1^. Plates were incubated at 37°C with shaking (180 rpm), and after 0, 2, and 4 h, samples were serially diluted 10-fold in PBS and plated onto TSA to enumerate surviving CFU mL^−1^.

### Statistical analyses.

CFU data were log_10_ transformed and presented as the geometric mean ± geometric standard deviation. Non-CFU data were presented as the mean ± standard deviation except MIC data, which were presented as the median value. All experiments consisted of at least three independent biological replicates and were analyzed as described in the figure legends using GraphPad Prism (v8.0).
