# Supplementary material for: XerC Is Required for the Repair of Antibiotic- and Immune-Mediated DNA Damage in Staphylococcus aureus
Source: Antimicrob Agents Chemother. 2023 Feb 21;67(3):e01206-22. doi: 10.1128/aac.01206-22 (PMC10019262; doi:10.1128/aac.01206-22)
Supplement: Supplemental file 1 — Supplemental material. Download aac.01206-22-s0001.pdf, PDF file, 1.0 MB [file aac.01206-22-s0001.pdf]

XerC is required for the repair of antibiotic- and immune-mediated DNA damage in *Staphylococcus aureus*

Elizabeth V. K. Ledger[1], Katie Lau[1], Edward W. Tate[2] and Andrew M. Edwards[1#]

Supplementary information

Supplementary Figures 1-2

Supplementary Tables 1-3

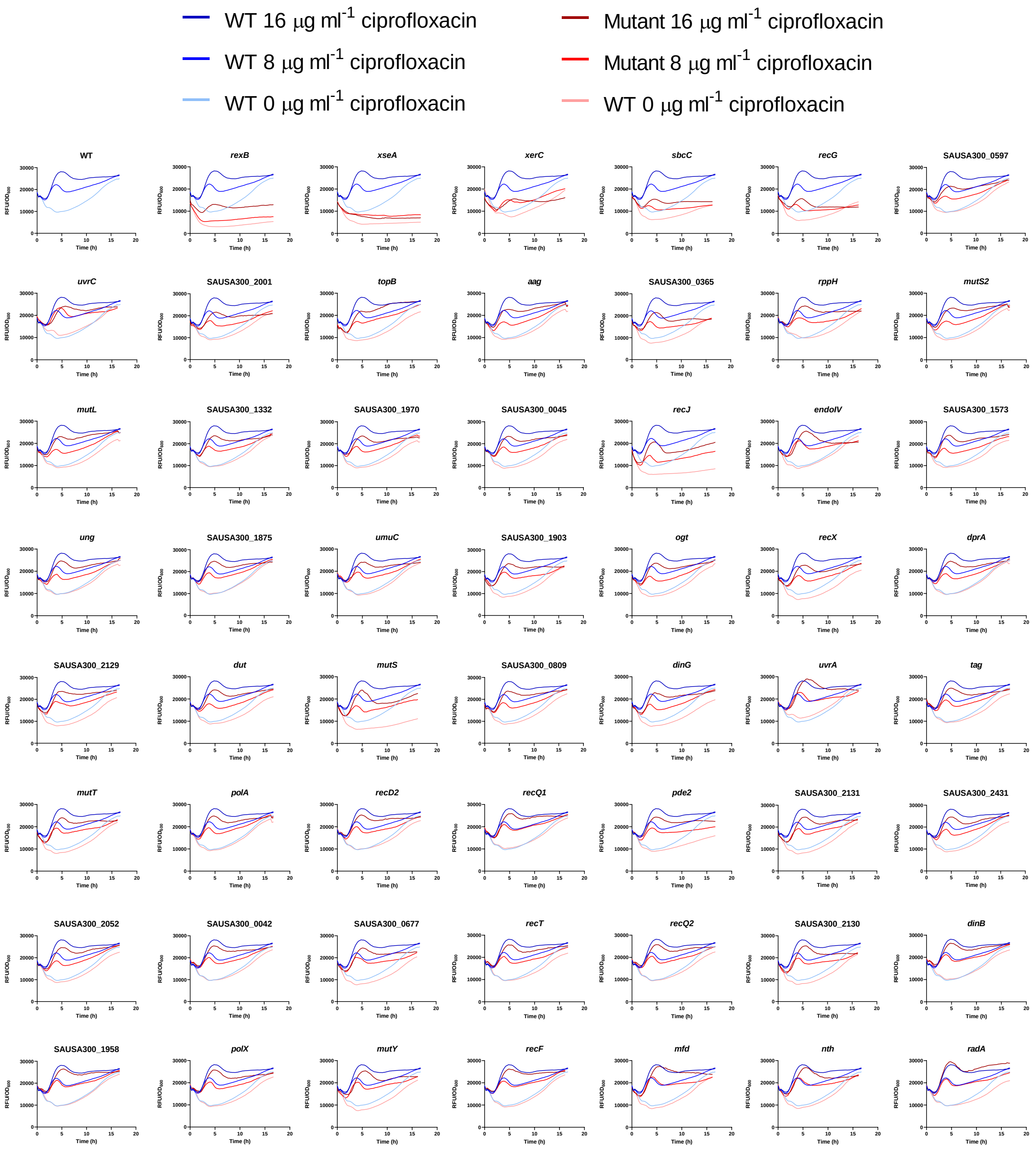

Fig. S1. SOS response of DNA repair mutants. USA300 *S. aureus* WT and NARSA mutants defective for various DNA repair genes containing the P*recA-gfp* reporter were exposed to 0, 8 or 16 µg ml^{-1} ciprofloxacin and GFP and OD_{600} measured over 17 h. GFP was divided by OD_{600} to normalise for changes in cell density which occurred during the assay.

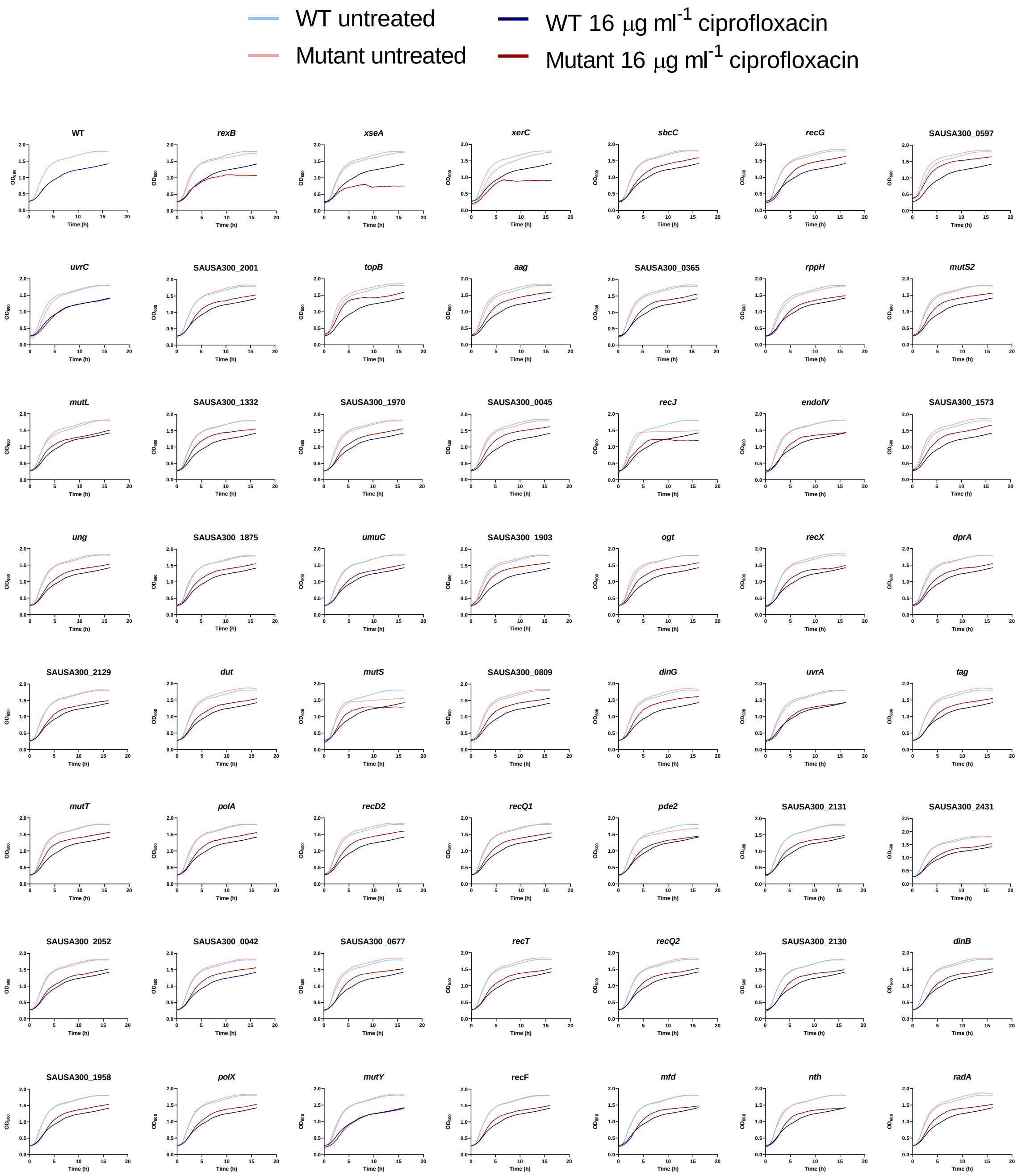

Fig. S2. Ciprofloxacin susceptibility of DNA repair mutants. USA300 *S. aureus* WT and NARSA mutants defective for various DNA repair genes containing the P*recA*-*gfp* reporter were exposed to 0 or 16 μg ml^{-1} ciprofloxacin and OD_{600} measured over 17 h.

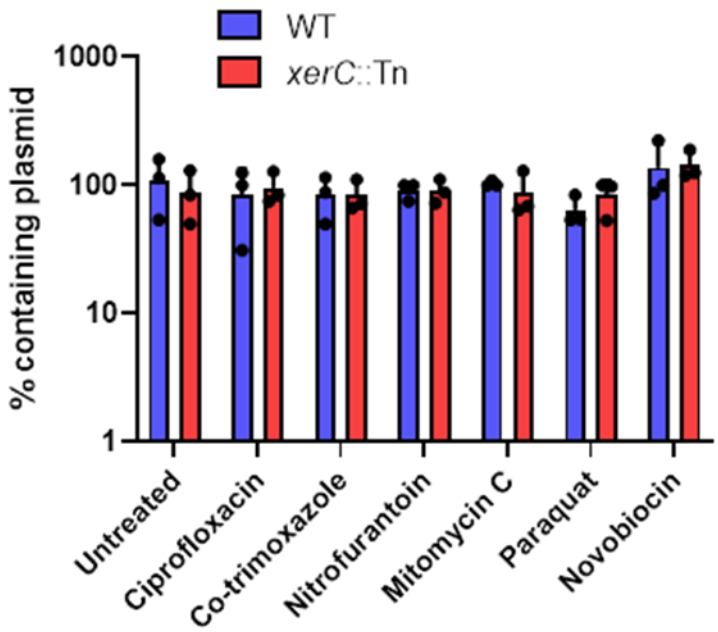

Fig. S3. Loss of reporter plasmid does not explain reduced SOS response. *S. aureus* USA300 JE2 WT containing the P*recA*-*gfp* reporter plasmid were exposed to no antibiotic, 32 µg ml^{-1} ciprofloxacin, 4 µg ml^{-1} co-trimoxazole, 32 µg ml^{-1} nitrofurantoin, 0.25 µg ml^{-1} mitomycin C, 16 µM paraquat or 0.25 µg ml^{-1} novobiocin for 16 h before being plated onto TSA or TSA supplemented with 90 µg ml^{-1} kanamycin and the percentage of colonies containing the reporter plasmid determined. Data represent the mean ± standard deviation of three independent experiments and were analysed by two-way ANOVA (no significant differences between WT and mutant under any condition).

Table S1. Mutants screened for ability to induce SOS response

NARSA reference	Gene	Gene name	Description
NE1012	SAUSA300_0869	*rexB*	Exonuclease RexB
NE458	SAUSA300_1472	*xseA*	Exodeoxyribonuclease VII, large subunit
NE1451	SAUSA300_1243	*sbcC*	Exonuclease
NE1344	SAUSA300_1120	*recG*	ATP-dependent DNA helicase
NE883	SAUSA300_1145	*xerC*	Tyrosine recombinase XerC
NE1878	SAUSA300_0597		Putative endonuclease III
NE1212	SAUSA300_1045	*uvrC*	Excinuclease ABC subunit C
NE93	SAUSA300_2001		Similar to DNA mismatch repair protein
NE152	SAUSA300_2208	*topB*	DNA topoisomerase III
NE1613	SAUSA300_2290	*aag*	Putative 3-methyladenine DNA glycosylase
NE1900	SAUSA300_0365		Hypothetical protein
NE746	SAUSA300_1734	*rppH*	Conserved hypothetical protein
NE1462	SAUSA300_1043	*mutS2*	DNA mismatch repair MutS2 protein
NE80	SAUSA300_1189	*mutL*	DNA mismatch repair protein MutL
NE789	SAUSA300_1332		Putative 5'-3' exonuclease
NE246	SAUSA300_1970		Putative exonuclease
NE88	SAUSA300_0045		HNH endonuclease family protein
NE11	SAUSA300_1592	*recJ*	ssDNA exonuclease
NE1028	SAUSA300_1517	*endoIV*	Endonuclease IV
NE1794	SAUSA300_1573		Holliday junction resolvase-like protein
NE888	SAUSA300_0563	*ung*	Uracil-DNA glycosylase
NE1320	SAUSA300_1875		Exonuclease
NE445	SAUSA300_1259	*umuC*	ImpB/MucB/SamB family protein
NE692	SAUSA300_1903		Conserved hypothetical protein
NE1907	SAUSA300_2485	*ogt*	Methylated DNA-protein cysteine methyltransferase
NE324	SAUSA300_1854	*recX*	Regulatory protein RecX
NE242	SAUSA300_1142	*dprA*	DNA protecting protein DprA
NE993	SAUSA300_2129		Putative hemolysin III (regulated by RecA)
NE1798	SAUSA300_1949	*dut*	dUTP diphosphatase
NE974	SAUSA300_1188	*mutS*	DNA mismatch repair protein mutS
NE466	SAUSA300_0809		Putative DNA primase
NE346	SAUSA300_1346	*dinG*	Putative DnaQ family exonuclease/DinG family helicase
NE145	SAUSA300_0742	*uvrA*	Excinuclease ABC, A subunit
NE1825	SAUSA300_1612	*tag*	DNA-3-methyladenine glycosidase
NE1653	SAUSA300_2432	*mutT*	MutT/NUDIX family hydrolase
NE22	SAUSA300_1636	*polA*	DNA polymerase I superfamily
NE1427	SAUSA300_1576	*recD2*	Helicase, RecD/TraA family
NE972	SAUSA300_0705	*recQ1*	ATP-dependent DNA helicase RecQ
NE1208	SAUSA300_1650	*pde2*	Hypothetical protein
NE1146	SAUSA300_2131		Hypothetical protein (regulated by RecA)
NE513	SAUSA300_2431		Putative helicase
NE1679	SAUSA300_2052		Single-stranded DNA- binding protein family
NE487	SAUSA300_0042		Conserved hypothetical protein
NE279	SAUSA300_0677	*phrB*	Putative DNA photolyase (regulated by RecA)
NE1830	SAUSA300_1960	*recT*	Putative phage-related DNA recombination protein
NE1528	SAUSA300_1371	*recQ2*	ATP-dependent DNA helicase RecQ
NE97	SAUSA300_2130		UTP-glucose-1-phosphate uridylyltransferase family protein (regulated by RecA)
NE1866	SAUSA300_1876	*dinB*	DNA polymerase IV
NE824	SAUSA300_1958		Single-strand binding protein
NE947	SAUSA300_1042	*polX*	Hypothetical protein
NE1040	SAUSA300_1849	*mutY*	A/G-specific adenine glycosylase
NE555	SAUSA300_0004	*recF*	DNA replication and repair protein RecF

NE188	SAUSA300_0481	*mfd*	Transcription-repair coupling factor
NE761	SAUSA300_1343	*nth*	Endonuclease III
NE1176	SAUSA300_0511	*radA*	DNA repair protein RadA

Table S2. Strains used in this study

Strain	Relevant characteristics	Reference/source
USA300 JE2 WT	LAC strain of the USA300 CA-MRSA lineage cured of plasmids	19
USA300 JE2 *xerC*::Tn	JE2 with a *bursa aurealis* transposon insertion in *xerC*, Ery^{r}	19
USA300 JE2 *xerC*::Tn p*itet-xerC*	JE2 with a *bursa aurealis* transposon insertion in *xerC* complemented with *pitet-xerC.* Ery^{r}*,* Cm^{r}	This study
USA300 JE2 *xerC*::Tn p*itet-xerC*^{Y273F}	JE2 with a *bursa aurealis* transposon insertion in *xerC* complemented with *pitet-xerC*^{Y273F}*.* Ery^{r}*,* Cm^{r}.	This study
USA300 JE2 WT P*recA_gfp*	USA300 LAC JE2 carrying the P*recA-gfp* reporter plasmid, Kan^{r}	11
USA300 JE2 *rexB*::Tn P*recA_gfp*	JE2 with a *bursa aurealis* transposon insertion in *rexB* carrying the P*recA-gfp* reporter plasmid, Kan^{r}, Ery^{r}*.*	11
USA300 JE2 *uvrA*::Tn P*recA_gfp*	JE2 with a *bursa aurealis* transposon insertion in *uvrA* carrying the P*recA-gfp* reporter plasmid, Kan^{r}, Ery^{r}	This study
USA300 JE2 *sbcC*::Tn P*recA_gfp*	JE2 with a *bursa aurealis* transposon insertion in *sbcC* carrying the P*recA-gfp* reporter plasmid, Kan^{r}, Ery^{r}	This study
USA300 JE2 *recF*::Tn P*recA_gfp*	JE2 with a *bursa aurealis* transposon insertion in *recF* carrying the P*recA-gfp* reporter plasmid, Kan^{r}, Ery^{r}	This study
USA300 JE2 *nth*::Tn P*recA_gfp*	JE2 with a *bursa aurealis* transposon insertion in *nth* carrying the P*recA-gfp* reporter plasmid, Kan^{r}, Ery^{r}	This study
USA300 JE2 *uvrC*::Tn P*recA_gfp*	JE2 with a *bursa aurealis* transposon insertion in *uvrC* carrying the P*recA-gfp* reporter plasmid, Kan^{r}, Ery^{r}	This study
USA300 JE2 *mutS*::Tn P*recA_gfp*	JE2 with a *bursa aurealis* transposon insertion in *mutS* carrying the P*recA-gfp* reporter plasmid, Kan^{r}, Ery^{r}	This study
USA300 JE2 *recJ*::Tn P*recA_gfp*	JE2 with a *bursa aurealis* transposon insertion in *recJ* carrying the P*recA-gfp* reporter plasmid, Kan^{r}, Ery^{r}	This study
USA300 JE2 *endoIV*::Tn P*recA_gfp*	JE2 with a *bursa aurealis* transposon insertion in *endoIV* carrying the P*recA-gfp* reporter plasmid, Kan^{r}, Ery^{r}	This study
USA300 JE2 *xerC*::Tn P*recA_gfp*	JE2 with a *bursa aurealis* transposon insertion in *xerC* carrying the P*recA-gfp* reporter plasmid, Kan^{r}, Ery^{r}	This study
USA300 JE2 *recG*::Tn P*recA_gfp*	JE2 with a *bursa aurealis* transposon insertion in *recG* carrying the P*recA-gfp* reporter plasmid, Kan^{r}, Ery^{r}	This study

USA300 JE2 *mutT*::Tn P*recA_gfp*	JE2 with a *bursa aurealis* transposon insertion in *mutT* carrying the P*recA-gfp* reporter plasmid, Kan^r, Ery^r	This study
USA300 JE2 *mutY*::Tn P*recA_gfp*	JE2 with a *bursa aurealis* transposon insertion in *mutY* carrying the P*recA-gfp* reporter plasmid, Kan^r, Ery^r	This study
USA300 JE2 SAUSA300_2130::Tn P*recA_gfp*	JE2 with a *bursa aurealis* transposon insertion in SAUSA300_2130 carrying the P*recA-gfp* reporter plasmid, Kan^r, Ery^r	This study
USA300 JE2 SAUSA300_0677::Tn P*recA_gfp*	JE2 with a *bursa aurealis* transposon insertion in SAUSA300_0677 carrying the P*recA-gfp* reporter plasmid, Kan^r, Ery^r	This study
USA300 JE2 SAUSA300_1903::Tn P*recA_gfp*	JE2 with a *bursa aurealis* transposon insertion in SAUSA300_1903 carrying the P*recA-gfp* reporter plasmid, Kan^r, Ery^r	This study
USA300 JE2 SAUSA300_2131::Tn P*recA_gfp*	JE2 with a *bursa aurealis* transposon insertion in SAUSA300_2131 carrying the P*recA-gfp* reporter plasmid, Kan^r, Ery^r	This study
USA300 JE2 SAUSA300_0365::Tn P*recA_gfp*	JE2 with a *bursa aurealis* transposon insertion in SAUSA300_0365 carrying the P*recA-gfp* reporter plasmid, Kan^r, Ery^r	This study
USA300 JE2 *mfd*::Tn P*recA_gfp*	JE2 with a *bursa aurealis* transposon insertion in *mfd* carrying the P*recA-gfp* reporter plasmid, Kan^r, Ery^r	This study
USA300 JE2 SAUSA300_2129::Tn P*recA_gfp*	JE2 with a *bursa aurealis* transposon insertion in SAUSA300_2129 carrying the P*recA-gfp* reporter plasmid, Kan^r, Ery^r	This study
USA300 JE2 *ogt*::Tn P*recA_gfp*	JE2 with a *bursa aurealis* transposon insertion in *ogt* carrying the P*recA-gfp* reporter plasmid, Kan^r, Ery^r	This study
USA300 JE2 *ung*::Tn P*recA_gfp*	JE2 with a *bursa aurealis* transposon insertion in *ung* carrying the P*recA-gfp* reporter plasmid, Kan^r, Ery^r	This study
USA300 JE2 *aag*::Tn P*recA_gfp*	JE2 with a *bursa aurealis* transposon insertion in *aag* carrying the P*recA-gfp* reporter plasmid, Kan^r, Ery^r	This study
USA300 JE2 SAUSA300_0809::Tn P*recA_gfp*	JE2 with a *bursa aurealis* transposon insertion in SAUSA300_0809 carrying the P*recA-gfp* reporter plasmid, Kan^r, Ery^r	This study
USA300 JE2 *polA*::Tn P*recA_gfp*	JE2 with a *bursa aurealis* transposon insertion in *polA* carrying the P*recA-gfp* reporter plasmid, Kan^r, Ery^r	This study
USA300 JE2 *dut*::Tn P*recA_gfp*	JE2 with a *bursa aurealis* transposon insertion in *dut* carrying the P*recA-gfp* reporter plasmid, Kan^r, Ery^r	This study
USA300 JE2 *recJ*::Tn P*recA_gfp*	JE2 with a *bursa aurealis* transposon insertion in *recJ* carrying the P*recA-gfp* reporter plasmid, Kan^r, Ery^r	This study
USA300 JE2 *mutS2*::Tn P*recA_gfp*	JE2 with a *bursa aurealis* transposon insertion in *mutS2* carrying the P*recA-gfp* reporter plasmid, Kan^r, Ery^r	This study
USA300 JE2 *pde2*::Tn P*recA_gfp*	JE2 with a *bursa aurealis* transposon insertion in *pde2* carrying the P*recA-gfp* reporter plasmid, Kan^r, Ery^r	This study

USA300 JE2 *recQ1*::Tn P*recA_gfp*	JE2 with a *bursa aurealis* transposon insertion in *recQ1* carrying the P*recA-gfp* reporter plasmid, Kan^r, Ery^r	This study
USA300 JE2 *recT*::Tn P*recA_gfp*	JE2 with a *bursa aurealis* transposon insertion in *recT* carrying the P*recA-gfp* reporter plasmid, Kan^r, Ery^r	This study
USA300 JE2 *rppH*::Tn P*recA_gfp*	JE2 with a *bursa aurealis* transposon insertion in *rppH* carrying the P*recA-gfp* reporter plasmid, Kan^r, Ery^r	This study
USA300 JE2 *recD2*::Tn P*recA_gfp*	JE2 with a *bursa aurealis* transposon insertion in *recD2* carrying the P*recA-gfp* reporter plasmid, Kan^r, Ery^r	This study
USA300 JE2 *recQ2*::Tn P*recA_gfp*	JE2 with a *bursa aurealis* transposon insertion in *recQ2* carrying the P*recA-gfp* reporter plasmid, Kan^r, Ery^r	This study
USA300 JE2 *polX*::Tn P*recA_gfp*	JE2 with a *bursa aurealis* transposon insertion in *polX* carrying the P*recA-gfp* reporter plasmid, Kan^r, Ery^r	This study
USA300 JE2 *mutL*::Tn P*recA_gfp*	JE2 with a *bursa aurealis* transposon insertion in *mutL* carrying the P*recA-gfp* reporter plasmid, Kan^r, Ery^r	This study
USA300 JE2 *dinG*::Tn P*recA_gfp*	JE2 with a *bursa aurealis* transposon insertion in *dinG* carrying the P*recA-gfp* reporter plasmid, Kan^r, Ery^r	This study
USA300 JE2 SAUSA300_2431::Tn P*recA_gfp*	JE2 with a *bursa aurealis* transposon insertion in SAUSA300_2431 carrying the P*recA-gfp* reporter plasmid, Kan^r, Ery^r	This study
USA300 JE2 SAUSA300_1573::Tn P*recA_gfp*	JE2 with a *bursa aurealis* transposon insertion in SAUSA300_1573 carrying the P*recA-gfp* reporter plasmid, Kan^r, Ery^r	This study
USA300 JE2 SAUSA300_0597::Tn P*recA_gfp*	JE2 with a *bursa aurealis* transposon insertion in SAUSA300_0597 carrying the P*recA-gfp* reporter plasmid, Kan^r, Ery^r	This study
USA300 JE2 SAUSA300_2502::Tn P*recA_gfp*	JE2 with a *bursa aurealis* transposon insertion in SAUSA300_2502 carrying the P*recA-gfp* reporter plasmid, Kan^r, Ery^r	This study
USA300 JE2 SAUSA300_1958::Tn P*recA_gfp*	JE2 with a *bursa aurealis* transposon insertion in SAUSA300_1958 carrying the P*recA-gfp* reporter plasmid, Kan^r, Ery^r	This study
USA300 JE2 *dinB*::Tn P*recA_gfp*	JE2 with a *bursa aurealis* transposon insertion in *dinB* carrying the P*recA-gfp* reporter plasmid, Kan^r, Ery^r	This study
USA300 JE2 *umuC*::Tn P*recA_gfp*	JE2 with a *bursa aurealis* transposon insertion in *umuC* carrying the P*recA-gfp* reporter plasmid, Kan^r, Ery^r	This study
USA300 JE2 *recX*::Tn P*recA_gfp*	JE2 with a *bursa aurealis* transposon insertion in *recX* carrying the P*recA-gfp* reporter plasmid, Kan^r, Ery^r	This study
USA300 JE2 SAUSA300_1875::Tn P*recA_gfp*	JE2 with a *bursa aurealis* transposon insertion in SAUSA300_1875 carrying the P*recA-gfp* reporter plasmid, Kan^r, Ery^r	This study
USA300 JE2 *dprA*::Tn P*recA_gfp*	JE2 with a *bursa aurealis* transposon insertion in *dprA* carrying the P*recA-gfp* reporter plasmid, Kan^r, Ery^r	This study

USA300 JE2 SAUSA300_2001::Tn P*recA_gfp*	JE2 with a *bursa aurealis* transposon insertion in SAUSA300_2001 carrying the P*recA-gfp* reporter plasmid, Kan^r, Ery^r	This study
USA300 JE2 *topB*::Tn P*recA_gfp*	JE2 with a *bursa aurealis* transposon insertion in *topB* carrying the P*recA-gfp* reporter plasmid, Kan^r, Ery^r	This study
USA300 JE2 SAUSA300_1332::Tn P*recA_gfp*	JE2 with a *bursa aurealis* transposon insertion in SAUSA300_1332 carrying the P*recA-gfp* reporter plasmid, Kan^r, Ery^r	This study
USA300 JE2 SAUSA300_1955::Tn P*recA_gfp*	JE2 with a *bursa aurealis* transposon insertion in SAUSA300_1955 carrying the P*recA-gfp* reporter plasmid, Kan^r, Ery^r	This study
USA300 JE2 SAUSA300_0042::Tn P*recA_gfp*	JE2 with a *bursa aurealis* transposon insertion in SAUSA300_0042 carrying the P*recA-gfp* reporter plasmid, Kan^r, Ery^r	This study
USA300 JE2 *radA*::Tn P*recA_gfp*	JE2 with a *bursa aurealis* transposon insertion in *radA* carrying the P*recA-gfp* reporter plasmid, Kan^r, Ery^r	This study
USA300 JE2 SAUSA300_1970::Tn P*recA_gfp*	JE2 with a *bursa aurealis* transposon insertion in SAUSA300_1970 carrying the P*recA-gfp* reporter plasmid, Kan^r, Ery^r	This study
USA300 JE2 SAUSA300_0045::Tn P*recA_gfp*	JE2 with a *bursa aurealis* transposon insertion in SAUSA300_0045 carrying the P*recA-gfp* reporter plasmid, Kan^r, Ery^r	This study
USA300 JE2 *tag*::Tn P*recA_gfp*	JE2 with a *bursa aurealis* transposon insertion in *tag* carrying the P*recA-gfp* reporter plasmid, Kan^r, Ery^r	This study
USA300 JE2 *xseA*::Tn P*recA_gfp*	JE2 with a *bursa aurealis* transposon insertion in *xseA* carrying the P*recA-gfp* reporter plasmid, Kan^r, Ery^r	This study

Table S3. Primers used in this study.

**Primer**	**Sequence (5' – 3') –** restriction sites underlined
*xerC*_Fw	ATGCCCTAGGGTATTGAATCATATTCAAGATGCG
*xerC*_Rev	CGATGTTTAAACGTATTACTCATGTTTCATTCTCC
*xerC*_Y273F_Fw	ACTGGTAAATTTACACACGTATC
*xerC*_Y273F_Rev	TGTTGACAAATTAACATGAC
